# Supplementary material for: Mechanical properties of native and decellularized reproductive tissues: insights for tissue engineering strategies
Source: Sci Rep. 2024 Mar 28;14:7347. doi: 10.1038/s41598-024-57867-5 (PMC10973341; doi:10.1038/s41598-024-57867-5)
Supplement: Supplementary file 3 — Supplementary Information 3. [file 41598_2024_57867_MOESM3_ESM.docx]

**Mechanical Properties of Native and Decellularized Reproductive Tissues: Insights for Tissue Engineering Strategies**

R. Franko^1,2§^, Y. Franko^1,2§^, E. Ribes Martinez^1,2§^, G.A. Ferronato^1,2§^, I. Heinzelmann^1,2§^, N. Grechi^1,2§^, S. Devkota^1,2§^, P.K. Fontes^3§^, R. Coeti^1,2§^, T.S.I. Oshiro^4§^, M.A.M.M. Ferraz^1,2*^

^1^Clinic of Ruminants, Faculty of Veterinary Medicine, Ludwig-Maximilians-Universität München, Sonnenstr. 16, Oberschleißheim, 85764, Germany

^2^Gene Center, Ludwig-Maximilians-Universität München, Feodor-Lynen Str. 25, Munich, 81377, Germany.

^3^Center of Natural and Human Sciences, Federal University of ABC, Av. dos Estados, 5001, Santo André, SP, 09210-580, Brazil

^4^Department of Surgery, Faculty of Veterinary Medicine and Zootechny, University of São Paulo, Av. Prof. Dr. Orlando Marques de Paiva, 87 Anatomy Building - University City, São Paulo, SP, 05508-270, Brazil.

^§^These authors contributed equally to this manuscript, see author’s contributions for details.

*Corresponding author: E-mail: [m.ferraz@lmu.de](mailto:m.ferraz@lmu.de)

**Supplementary files**

***Validation of decellularization method***

Following the implementation of a sequential decellularization protocol involving sodium deoxycholate and DNase I, the effectiveness of the employed method was validated through the application of Hoechst 33342 nuclear staining to evaluate the decellularized and native tissues (Suppl. Fig. 1a illustrates the comprehensive results of the decellularization process). To quantify the depletion of DNA in the samples subsequent to decellularization, DNA extraction was performed prior to and after the procedure. Analysis of the obtained data exhibited a remarkable DNA removal of 97.0, 97.6, 96.6, and 96.3% for endometrium, ovary, oviduct, and testis, respectively, during the decellularization process (Suppl. Fig. 1b).


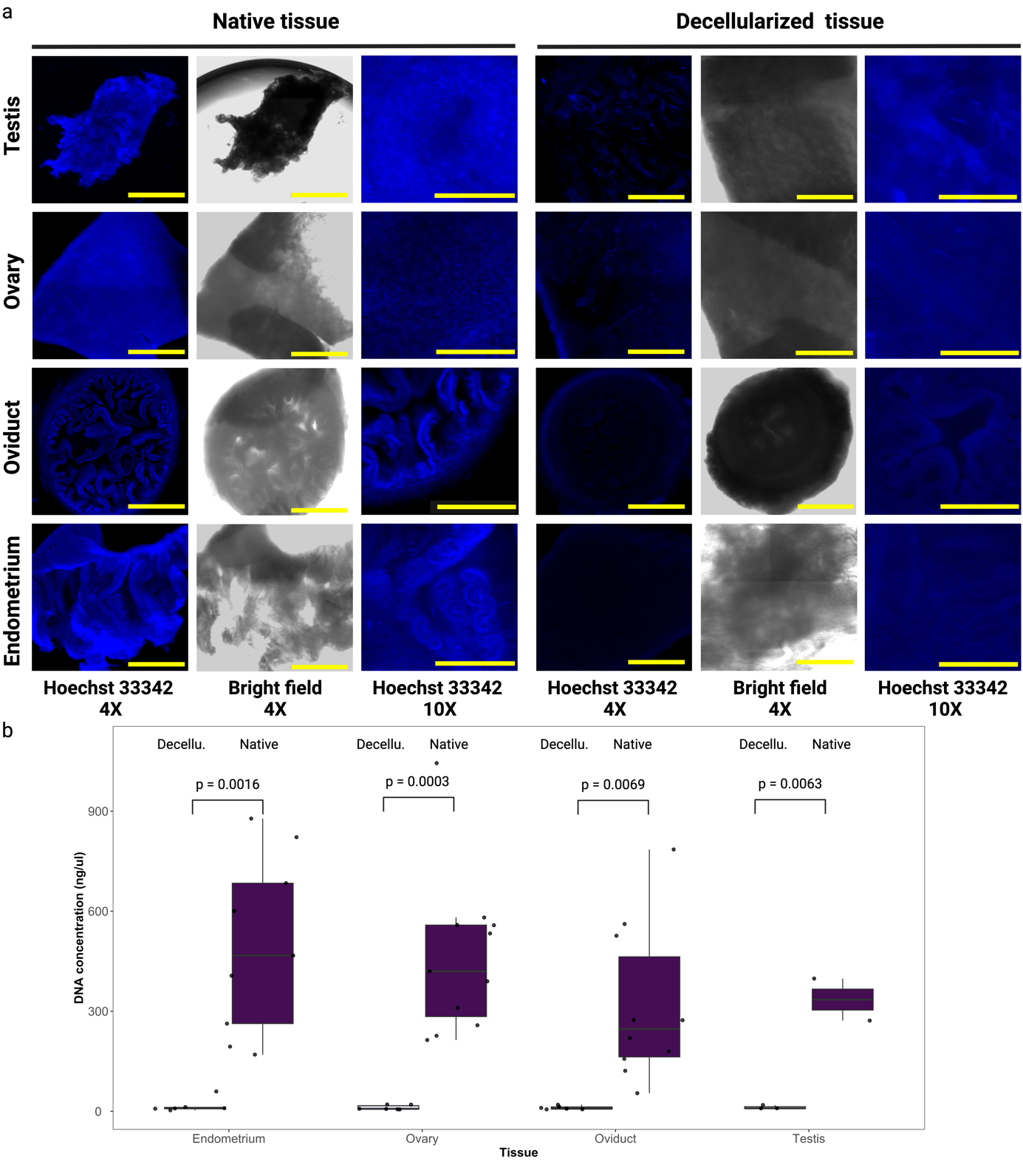


**Supplementary Figure 1.** Validation of decellularization protocol. Representative images of nuclear staining (Hoechst 33342) of native and decellularized tissues are shown (**a**). Decellularization efficiency was also validated by DNA (**b**) quantification of native and decellularized tissues. Scale bar for 4x images = 2,000 µm. Scale bar for 10x images = 500 µm.


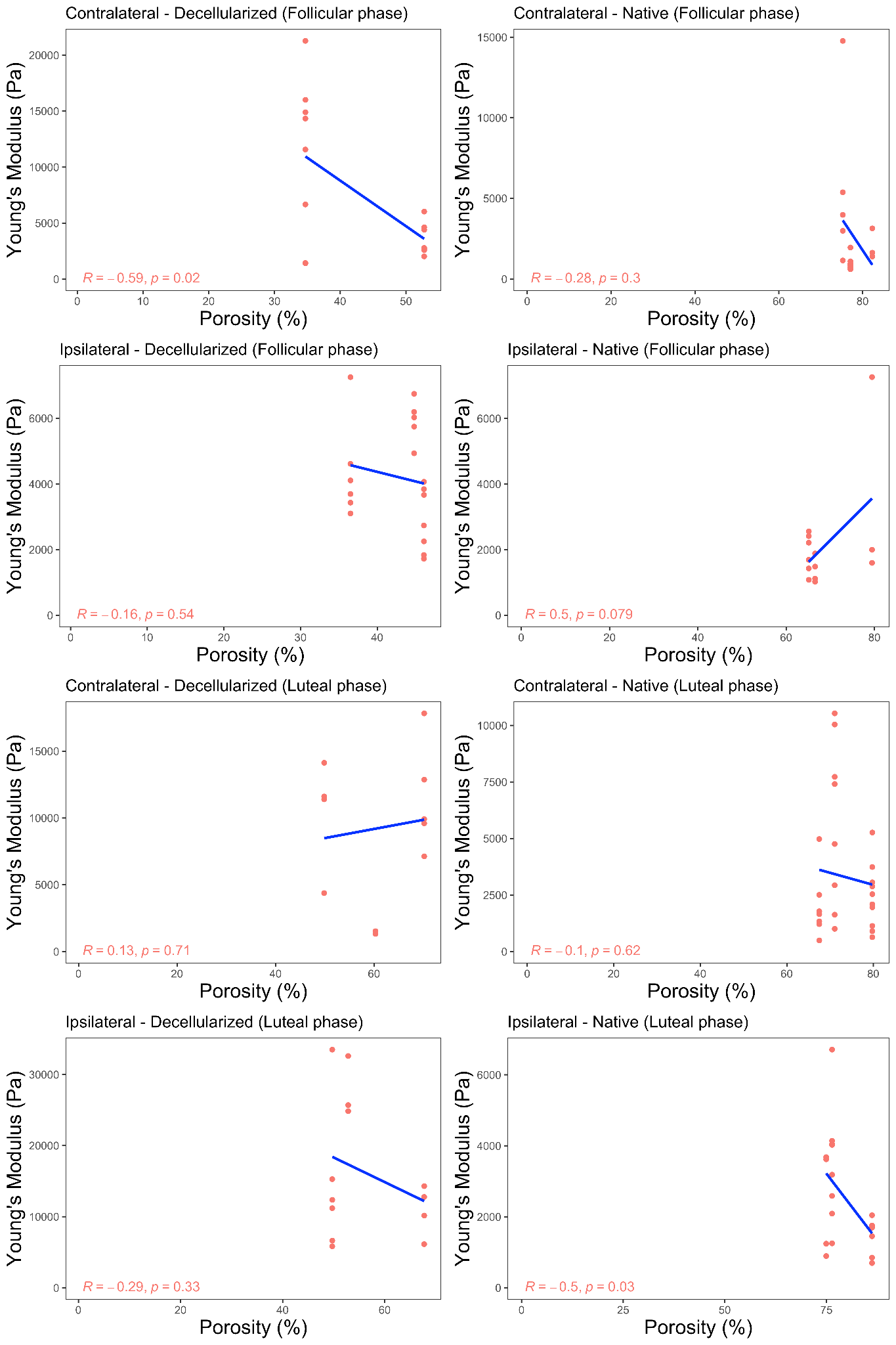


**Supplementary Figure 2.** Correlation plots of porosity and Young’s modulus in endometrium tissues. Analysis was performed in native and decellularized tissues collected from ipsi- and contralateral horns of endometrium tissues from cows at luteal (n = 3 cows) and follicular (n = 3 cows) phases of the estrous cycle


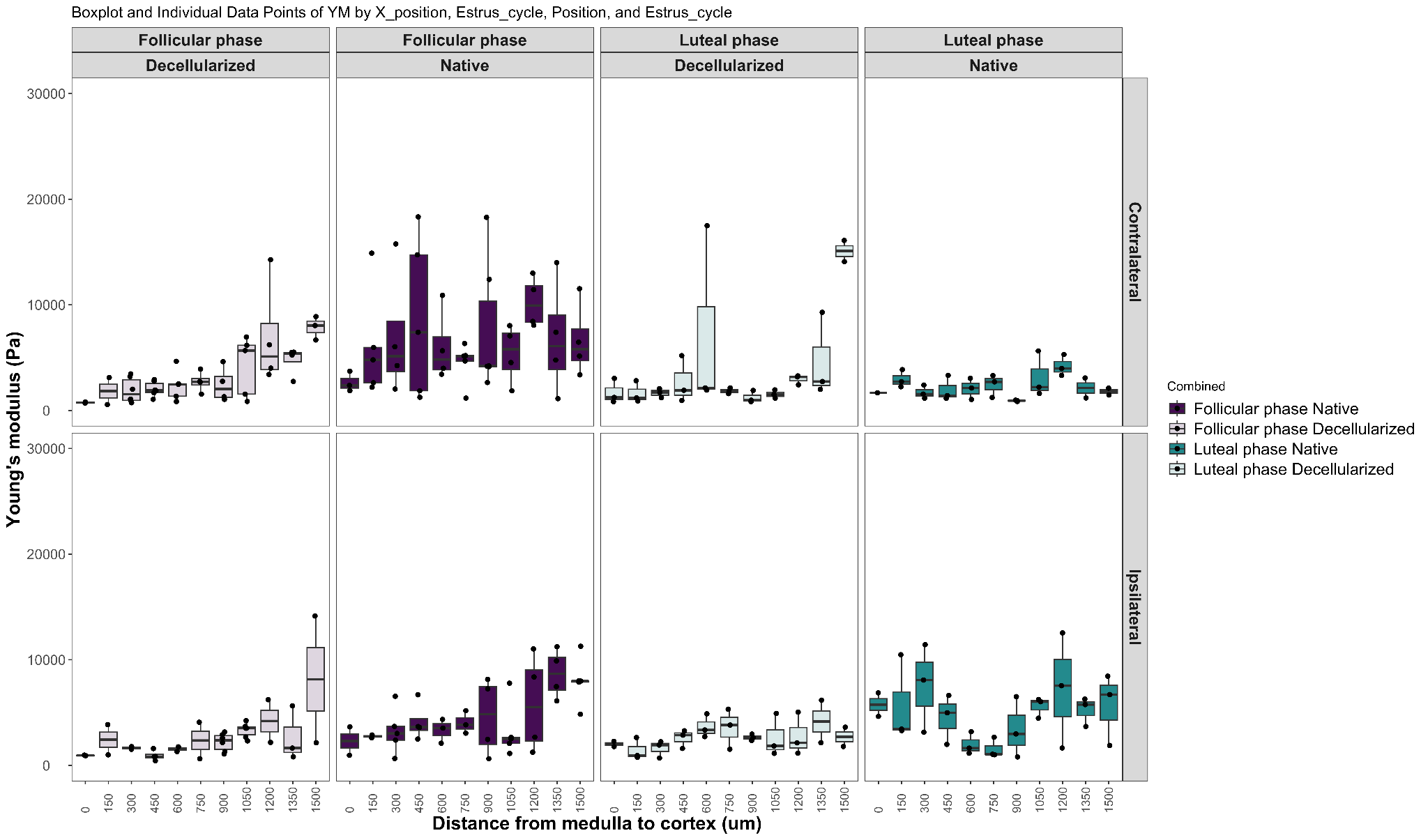


**Supplementary Figure 3.** Young’s modulus analysis in native and decellularized ovaries through nanoindentation specific distance points. After a transversal cut, the ovary tissues were mounted on a petri dish using 8 mg mL^-1^ agarose solution as a single piece, where the nanoindentation analysis was performed in a straightness acquisition mode with a 150 μm distance between analysis points, resulting in 1,500 μm x-axis distance in total, which 0 μm was the most external point and 1,500 μm the most inner. Analysis was performed in native and decellularized tissues collected from ipsi- and contralateral ovaries from cows at luteal (n = 3 cows) and follicular (n = 3 cows) phases of the estrous cycle.


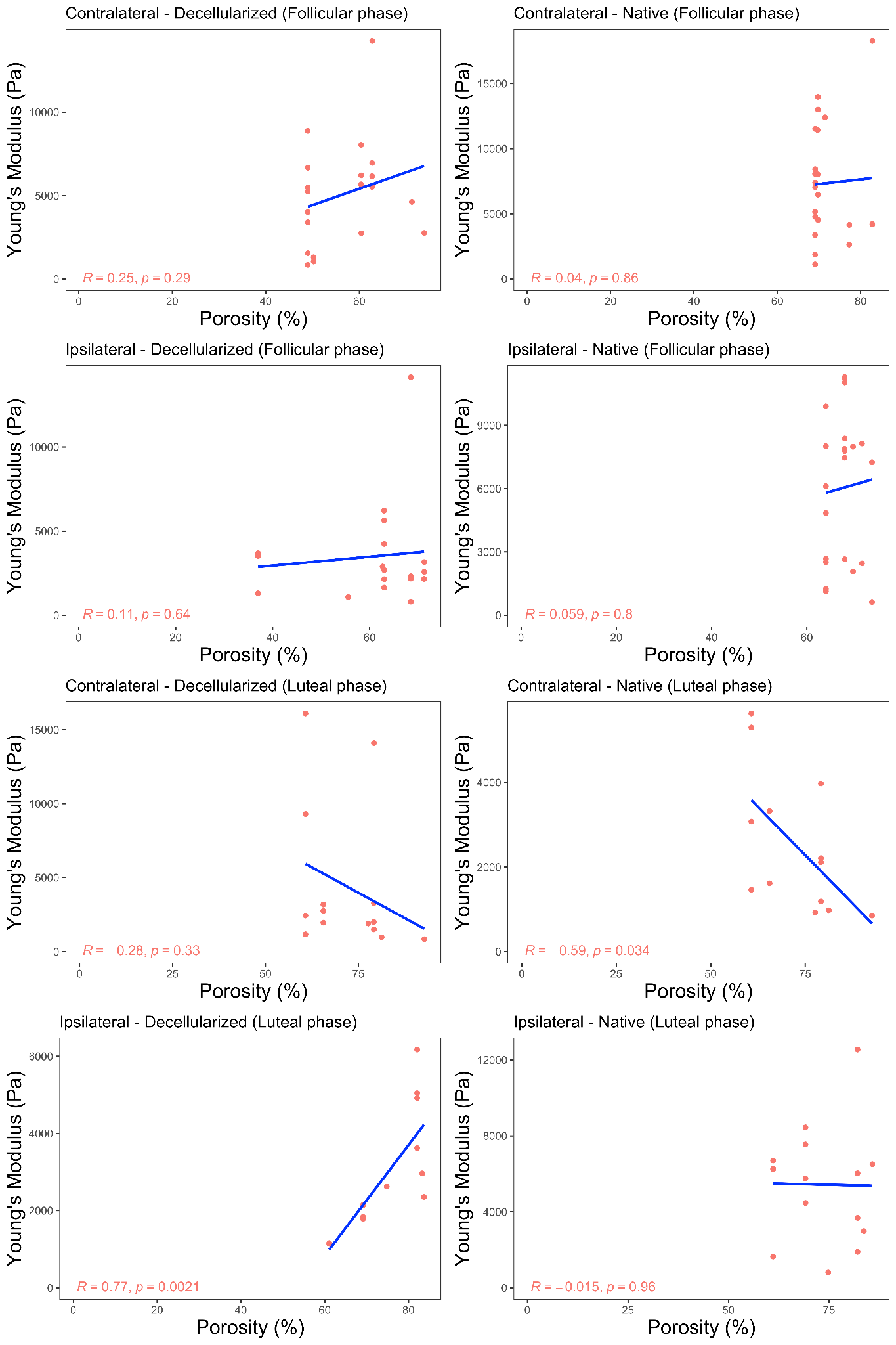


**Supplementary Figure 4.** Correlation plots of porosity and Young’s modulus in the cortical segment of the ovary. Analysis was performed in native and decellularized tissues collected from ipsi- and contralateral ovaries from cows at luteal (n = 3 cows) and follicular (n = 3 cows) phases of the estrous cycle.


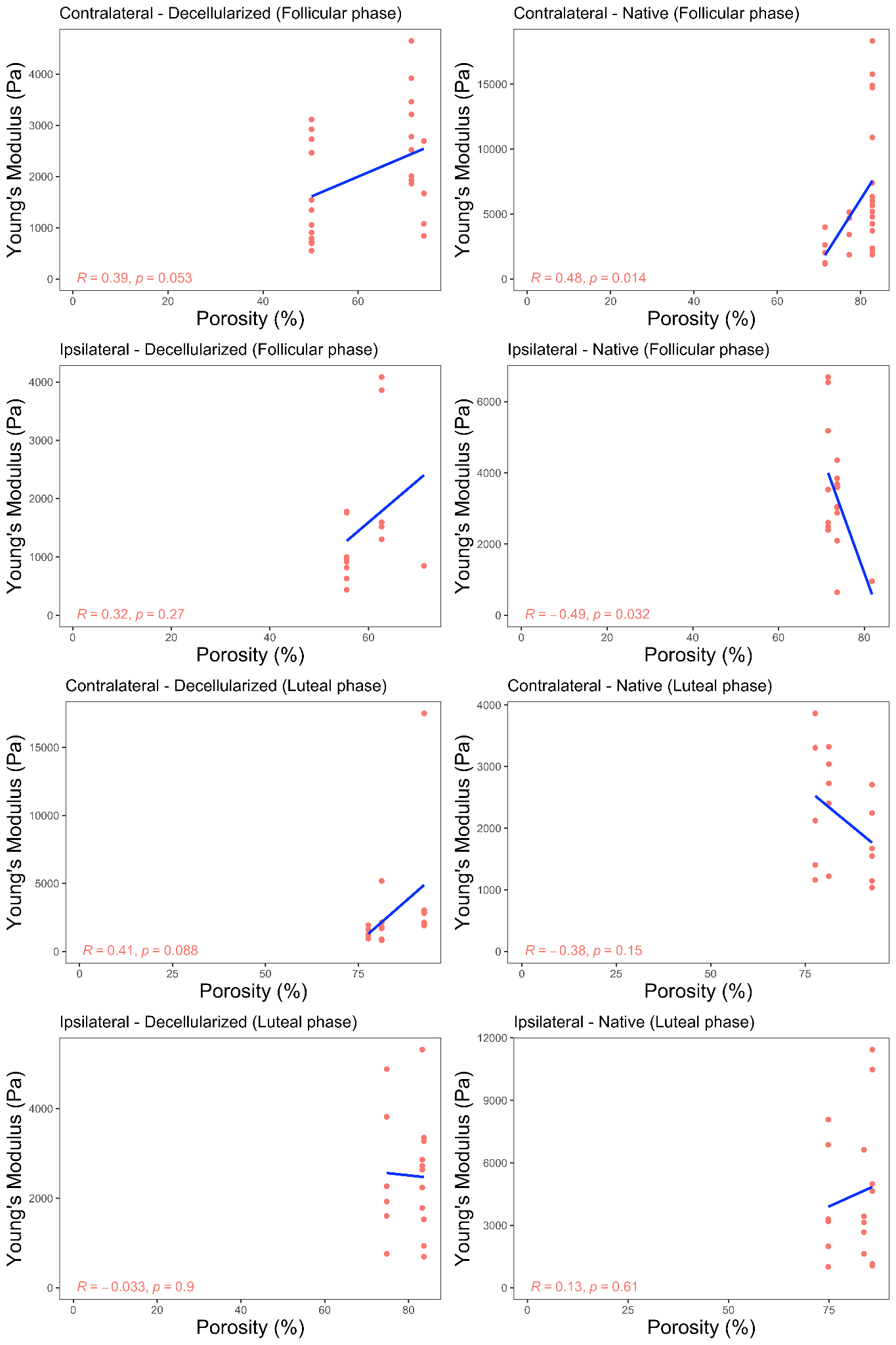


**Supplementary Figure 5.** Correlation plots of porosity and Young’s modulus in the medullar segment of the ovary. Analysis was performed in native and decellularized tissues collected from ipsi- and contralateral ovaries from cows at luteal (n = 3 cows) and follicular (n = 3 cows) phases of the estrous cycle.


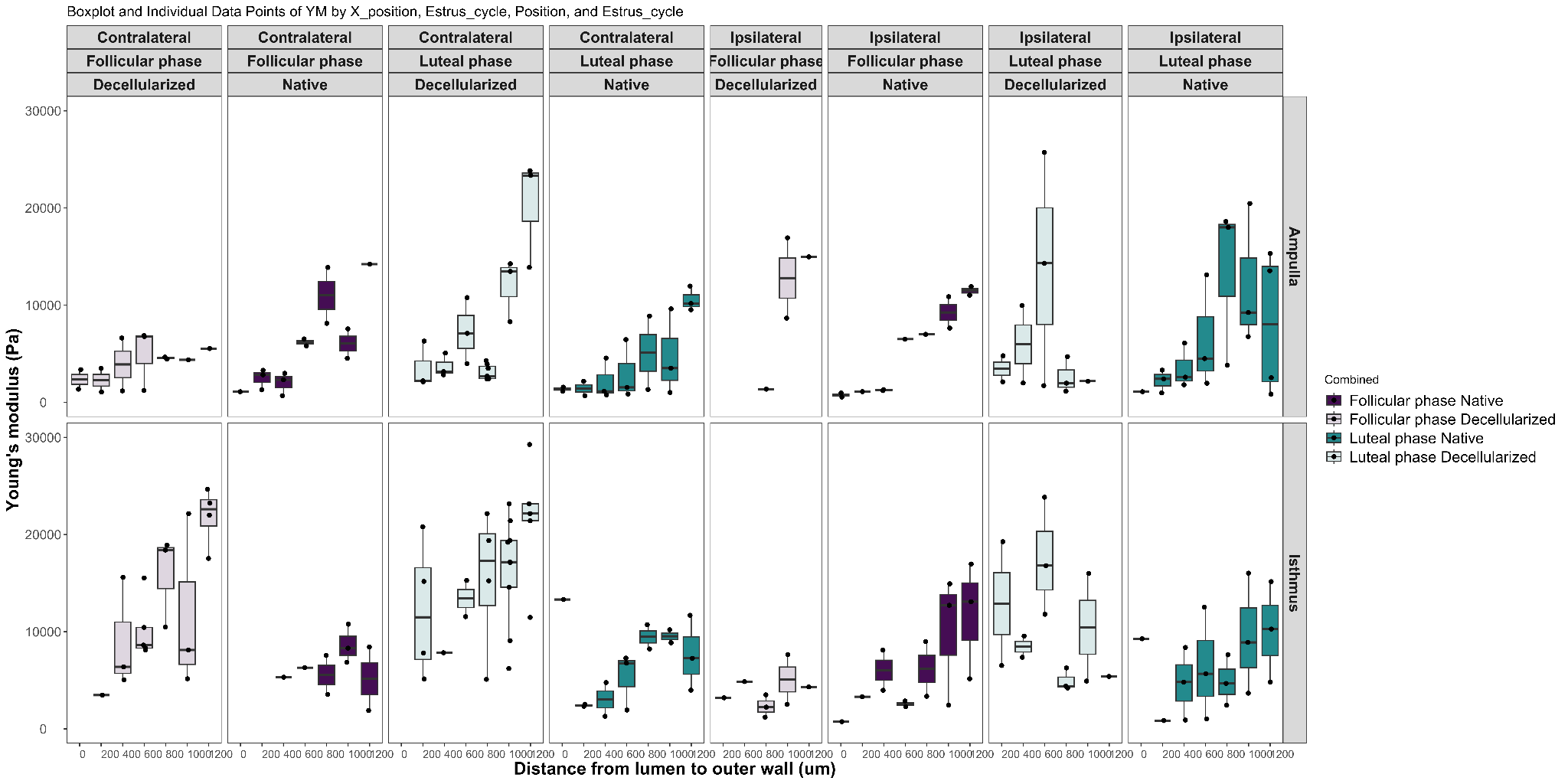


**Supplementary Figure 6.** Young’s modulus analysis in native and decellularized oviductal samples through nanoindentation specific distance points. After a transversal cut, the oviductal tissues were longitudinally opened and mounted sideway on a petri dish using 8 mg mL^-1^ agarose solution, this way all the tissue’s layers were free for nanoindentation. For nanoindentor analysis, a straightness acquisition mode with a 200 μm distance between analysis points was performed, resulting in 1,200 μm x-axis distance in total, which 0 μm was the most internal point (luminal oviductal side) and 1,200 μm the most external (tunica oviductal side). Analysis was performed in native and decellularized tissues collected from ipsi- and contralateral ovaries from cows at luteal (n = 3 cows) and follicular (n = 3 cows) phases of the estrous cycle.


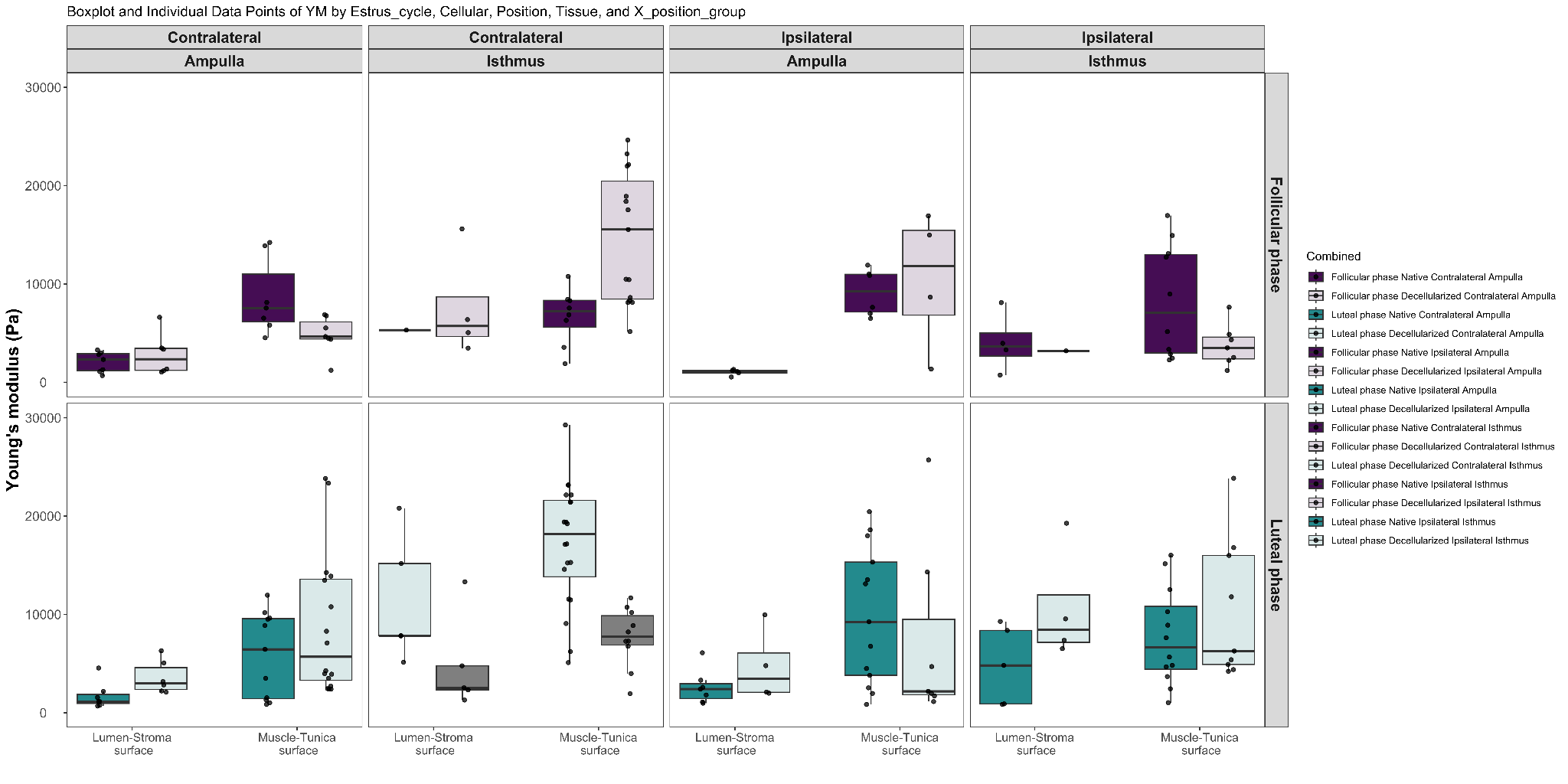


**Supplementary Figure 7.** Young’s modulus analysis in native and decellularized oviductal samples derived from supplementary figure 5. All the specific distance points presented in Supplementary Figure 5 were divided into two segments with 400 μm as threshold, which points lower than 400 μm were classified as Lumen-Stroma surface, and values higher than it was classified as Muscle-Tunica surface. Analysis was performed in native and decellularized tissues collected from ipsi- and contralateral ovaries from cows at luteal (n = 3 cows) and follicular (n = 3 cows) phases of the estrous cycle.


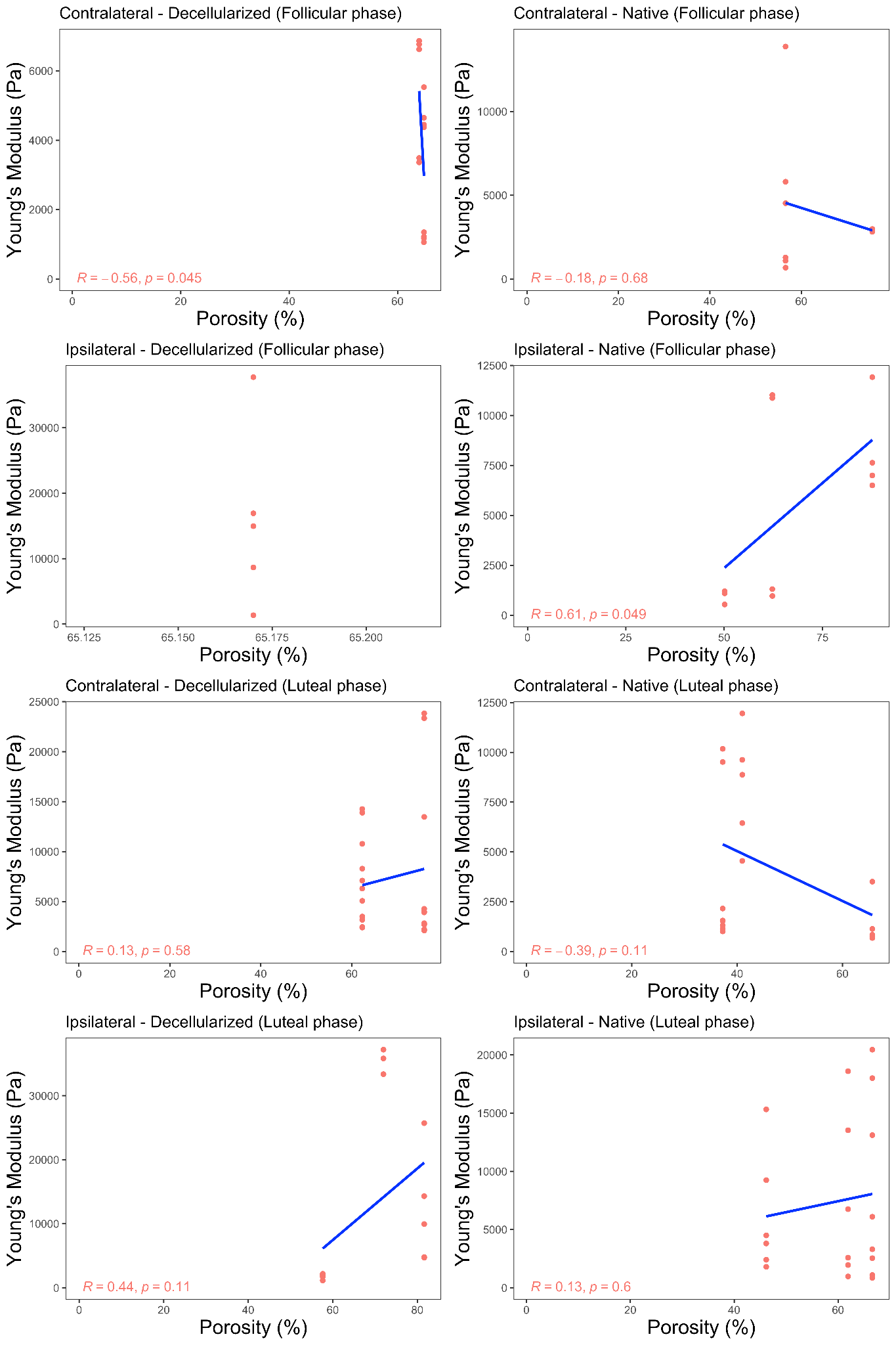


**Supplementary Figure 8.** Correlation plots of porosity and Young’s modulus in the ampullary segment of the oviduct. Analysis was performed in native and decellularized tissues collected from ipsi- and contralateral oviducts from cows at luteal (n = 3 cows) and follicular (n = 3 cows) phases of the estrous cycle.


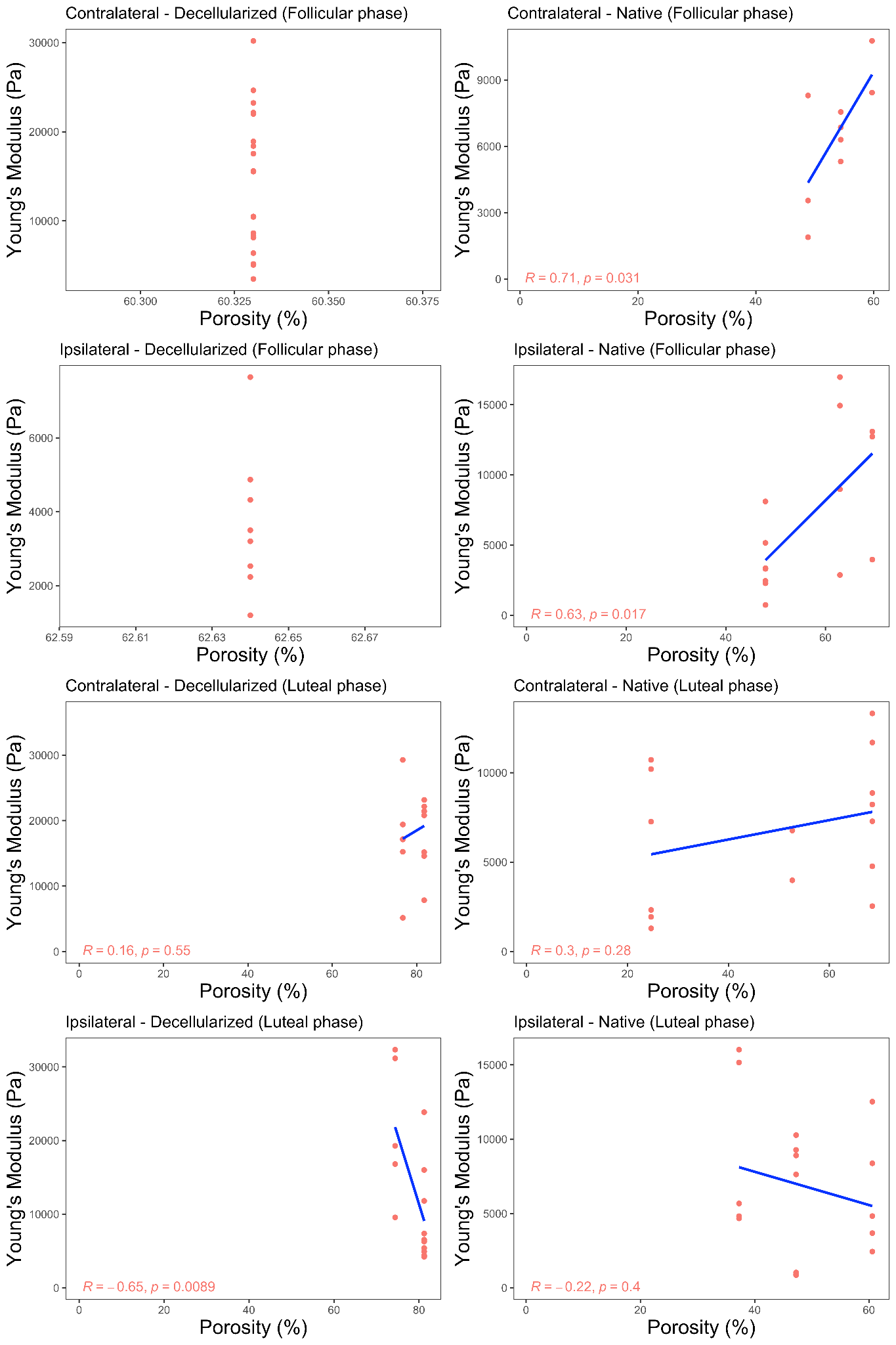


**Supplementary Figure 9.** Correlation plots of porosity and Young’s modulus in the isthmic segment of the oviduct. Analysis was performed in native and decellularized tissues collected from ipsi- and contralateral oviducts from cows at luteal (n = 3 cows) and follicular (n = 3 cows) phases of the estrous cycle.


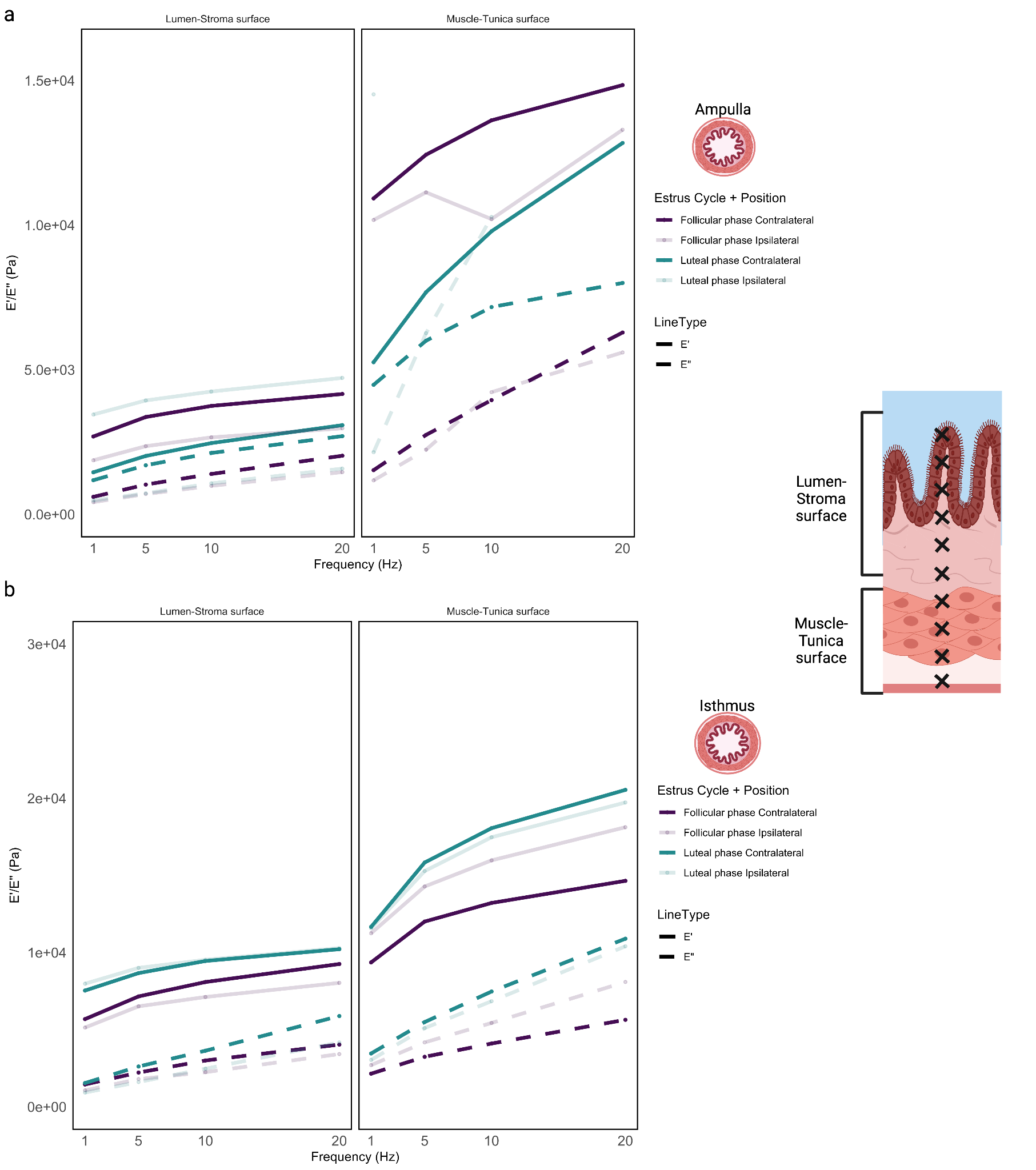


**Supplementary Figure 10.** Elastic and visco-elastic properties of the oviduct differentiated by specific distance points presented in Supplementary Figure 5 . Storage (E', continuous lines) and Loss (E", dashed lines) modulus of native oviductal ampulla (**a**) and isthmus (**b**). Oviduct layers were divided as Lumen-Stroma surface and Muscle-Tunica surface, and samples were analyzed for both follicular and luteal phases and ipsi- and contralateral positions.


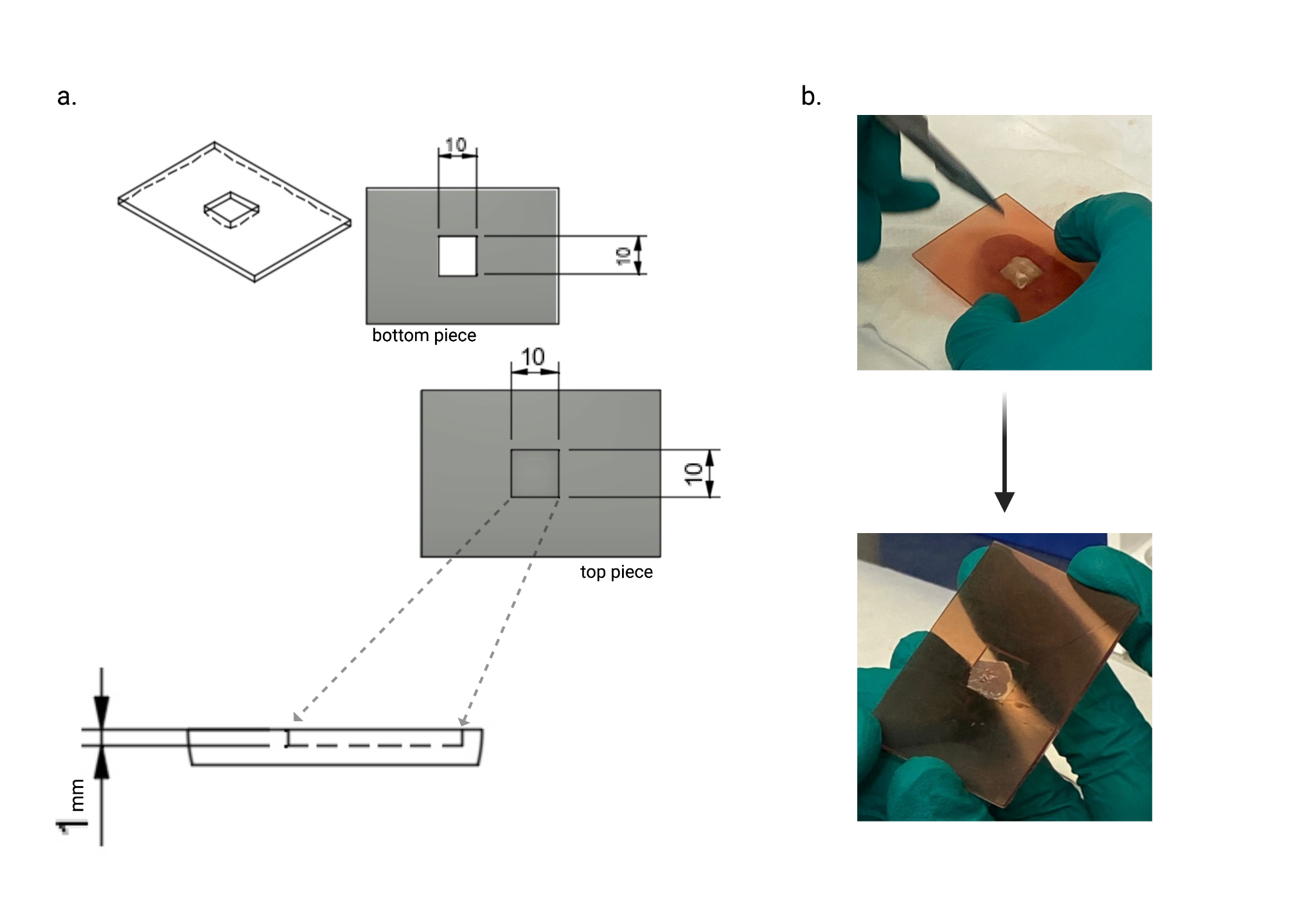


**Supplementary Figure 11.** Ovary slicer 3D print model (a) and example of the printed construct with sliced cortex (b).
